# Dual Functionality of HIV-1 Vif in APOBEC3 Counteraction and Cell Cycle Arrest

**DOI:** 10.3389/fmicb.2020.622012

**Published:** 2021-01-12

**Authors:** Daniel J. Salamango, Reuben S. Harris

**Affiliations:** ^1^Department of Biochemistry, Molecular Biology and Biophysics, University of Minnesota, Minneapolis, MN, United States; ^2^Masonic Cancer Center, University of Minnesota, Minneapolis, MN, United States; ^3^Institute for Molecular Virology, University of Minnesota, Minneapolis, MN, United States; ^4^Howard Hughes Medical Institute, University of Minnesota, Minneapolis, MN, United States

**Keywords:** APOBEC3, APOBEC3G, cell cycle arrest, PPP2R5, Vif

## Abstract

Accessory proteins are a key feature that distinguishes primate immunodeficiency viruses such as human immunodeficiency virus type I (HIV-1) from other retroviruses. A prime example is the virion infectivity factor, Vif, which hijacks a cellular co-transcription factor (CBF-β) to recruit a ubiquitin ligase complex (CRL5) to bind and degrade antiviral APOBEC3 enzymes including APOBEC3D (A3D), APOBEC3F (A3F), APOBEC3G (A3G), and APOBEC3H (A3H). Although APOBEC3 antagonism is essential for viral pathogenesis, and a more than sufficient functional justification for Vif’s evolution, most viral proteins have evolved multiple functions. Indeed, Vif has long been known to trigger cell cycle arrest and recent studies have shed light on the underlying molecular mechanism. Vif accomplishes this function using the same CBF-β/CRL5 ubiquitin ligase complex to degrade a family of PPP2R5 phospho-regulatory proteins. These advances have helped usher in a new era of accessory protein research and fresh opportunities for drug development.

## Introduction

It has been nearly 40 years since the discovery that acquired immune deficiency syndrome (AIDS) is caused by a retrovirus named human immunodeficiency virus type I (HIV-1) (Barré-Sinoussi et al., 1983; Gallo et al., 1984; Popovic et al., 1984). AIDS arises from a profound state of immune suppression brought on by a massive depletion of CD4^+^ T lymphocytes following HIV-1 infection, which results in patients succumbing to opportunistic infections and rare malignancies (Klatzmann et al., 1984; Masur et al., 1989; Ho et al., 1995). Soon after its discovery, the HIV-1 genome was sequenced and found to contain nine open reading frames that encode over a dozen viral proteins essential for pathogenicity (Emerman and Malim, 1998; Frankel and Young, 1998; Malim and Emerman, 2008).

**FIGURE 1:**
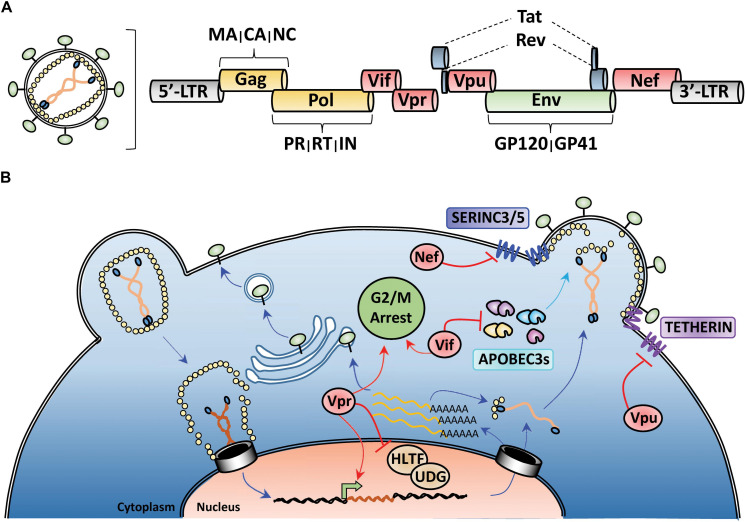
HIV-1 genome organization and counteraction of host-cell restriction factors by viral accessory proteins. **(A)** Depiction of the genomic organization of HIV-1 with yellow indicating structural proteins and viral enzymes, green indicating the envelope glycoprotein, blue indicating the regulatory proteins, and red highlighting the viral accessory proteins. **(B)** Simplified schematic of the HIV-1 life-cycle with activities of the viral accessory proteins highlighted in red. Nef and Vpu downmodulate the anti-viral factors TETHERIN and SERINC3/5 from the cell surface. Vif targets cytoplasmic APOBEC3s for proteasomal degradation prior to viron encapsidation. Vpr antagonizes many different cellular processes including DNA-damage response, cell cycle progression, and viral gene expression.

All retroviruses encode Gag, Pol, and Env polyproteins that are processed by viral and cellular proteases to release individual proteins during nascent particle assembly. Gag encodes structural proteins matrix (MA), capsid (CA), and nucleocapsid (NC), Pol encodes the viral enzymes protease (PR), reverse-transcriptase (RT), and integrase (IN), and Env encodes the surface glycoprotein GP160, which is processed into the receptor-binding GP120 and transmembrane GP41 domains by a cellular furin-like protease (Figure 1A). Lentiviruses encode additional proteins, such as the regulatory proteins Tat and Rev and several accessory proteins (Vif, Vpr, Vpu, and Nef) that perform various functions to enhance HIV-1 pathogenicity (Emerman and Malim, 1998; Malim and Emerman, 2008; Figures 1A,B). In at least three instances, the major function of these accessory proteins is to promote HIV-1 evasion of cell-intrinsic antiviral resistance by either re-localizing antiviral factors from their sites of virus restriction (Vpu-TETHERIN), targeting them for proteasomal degradation (Vif-APOBEC3), or a combination of both activities (Nef-SERINC3/5) (Madani and Kabat, 1998; Simon et al., 1998a; Sheehy et al., 2002; Neil et al., 2008; Van Damme et al., 2008; Rosa et al., 2015; Usami et al., 2015; Figure 1B). In the case of SERINC3/5 counteraction, Nef re-targets these proteins from the plasma membrane to endosomal/lysosomal compartments for degradation (Usami et al., 2015; Rosa et al., 2015; Shi et al., 2018). For Vpr, its role in HIV-1 pathogenesis remains enigmatic, with reported activities ranging from manipulation of the DNA-damage response (Hrecka et al., 2016; Wu et al., 2016) and induction of G2/M cell cycle arrest (Bartz et al., 1996; Sakai et al., 2006), to facilitating nuclear import of the HIV-1 genome (Heinzinger et al., 1994; Jenkins et al., 1998) and enhancing viral gene expression (Thierry et al., 2004; Zhang and Bieniasz, 2020; Figure 1B).

As is true for other viruses (e.g., herpesviruses and poxviruses), immunomodulatory accessory genes are frequently deleted, or dispensable, for viral replication *in vitro*; however, *in vivo* they are strongly maintained during natural infections. For example, *in vitro*, Vif, Vpr, Vpu, and Nef exert functions in a cell-type dependent manner, where these genes are dispensable for producing infectious viral particles in some T cell and myeloid cell lines but not others (i.e., permissive vs. non-permissive cells). However, in physiologically relevant primary CD4^+^ T cells and monocytes, loss of these genes results in severe impairment of HIV-1 replication (Deacon et al., 1995; Gaddis et al., 2004; Schindler et al., 2006). To further emphasize this point, HIV-1 deficient in the *vif* gene produces particles that are roughly 1000 times less infectious compared to wild-type virus in primary and non-permissive CD4^+^ T cells (Fisher et al., 1987; Strebel et al., 1987; Gabuzda et al., 1992).

## Discovery of Vif Function in APOBEC3 Degradation

The underlying mechanism of how Vif enhanced HIV-1 pathogenesis remained elusive for many years following its identification. The key to uncovering Vif’s function originated from observations that in some adherent (HeLa and HEK293T) and T cell lines (CEM-SS and SupT1) Vif is dispensable for producing infectious virus. In contrast, loss of Vif in physiologically relevant primary CD4^+^ T cells and macrophages, and some immortalized T cell lines (CEM and H9), results in the production of almost no infectious virus (Gabuzda et al., 1992; Sakai et al., 1993; Simon et al., 1998b). Comprehensive interrogation of these cell lines using heterokaryon experiments in parallel with subtraction cloning methods indicated the presence of a dominant factor, rather than the lack of a recessive gene product, and led to the identification of the host factor CEM15, now known as APOBEC3G (Madani and Kabat, 1998; Simon et al., 1998a; Sheehy et al., 2002).

**FIGURE 2:**
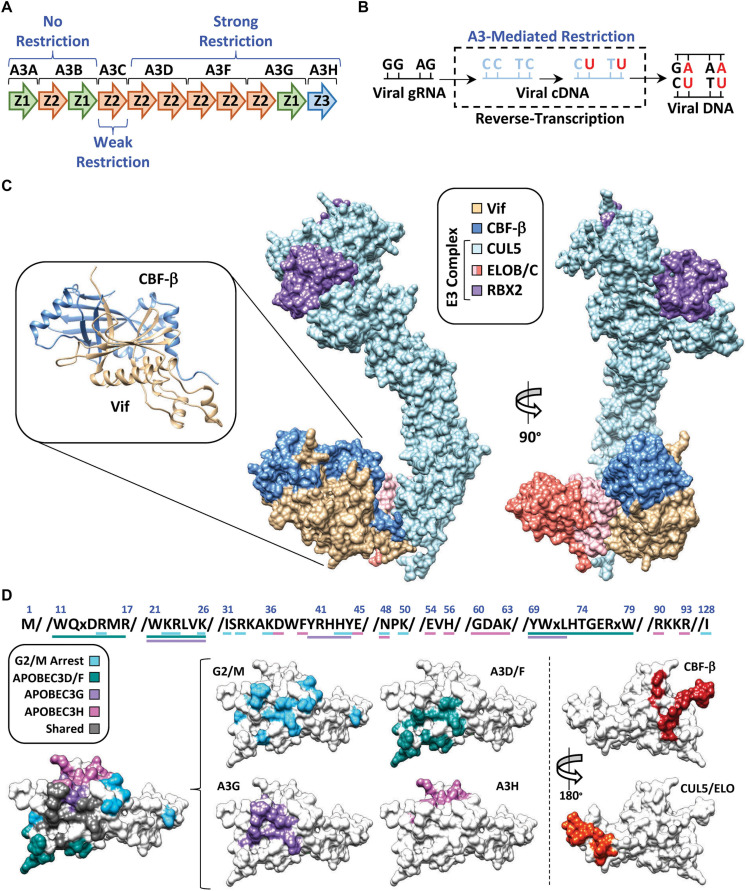
APOBEC3 restriction and counter action by Vif. **(A)** Schematic of the *APOBEC3* locus with anti-HIV-1 activity indicated above or below the respective APOBEC3 enzyme. **(B)** Depiction of APOBEC3 C-to-T mutations during conversion of the viral genomic RNA (gRNA) to cDNA. Mutations are depicted at either “CC” or “TC” dinucleotide contexts which is reflective of A3G or A3D/F/H target sites, respectively. Fixation of the mutations is depicted by the final double-stranded DNA product (right). **(C)** Structure of the E3-ubiquitin ligase complex nucleated by Vif (PDB: 4N9F). Full length CUL5 has been modeled in using the partial CUL5 sequence from PDB: 4N9F. RBX2 has been modeled in using RBX1 from PDB: 1LDJ as a template. The inset depicts the Vif/CBF-β hetero-dimer as ribbon diagrams from the crystal structure. **(D)** Amino acid sequences and surface models of Vif with substrate binding residues highlighted in the indicated color. The shared surface indicated residues that are required for degradation of two or more Vif substrates.

APOBEC3G (A3G) belongs to a family of cytosine deaminase enzymes that converts cytosine to uracil (C-to-U) in single-stranded DNA (Harris et al., 2002; Petersen-Mahrt et al., 2002; Figures 2A,B). DNA C-to-U deamination is catalyzed by a zinc-mediated hydrolysis reaction driven by a highly conserved glutamic acid residue located within the substrate-binding pocket. APOBEC3 enzymes exhibit an intrinsic dinucleotide preference with the cytosine base being preceded by either a thymine (TC) or another cytosine (CC) (Carpenter et al., 2010; Kohli et al., 2010; Rathore et al., 2013; Figure 2B). Because of its enzymatic classification, researchers postulated that A3G was able to restrict HIV-1 replication through deamination of the viral RNA during reverse-transcription, when the genome is converted to single-stranded DNA (Harris et al., 2003; Mangeat et al., 2003; Zhang et al., 2003; Di Noia and Neuberger, 2007). In fact, extensive biochemical characterization of A3G and related family members revealed that these enzymes could package into nascent HIV-1 particles and inflict catastrophic levels of C-to-T mutations in the viral genome during its conversion to single-stranded DNA (accounts for G-to-A mutations observed on the genomic plus strand) (Bishop et al., 2004; Desimmie et al., 2014; Feng et al., 2014; Figures 2A,B). In addition, several APOBEC3 enzymes also exert deaminase-independent antiviral activities and hinder reverse transcription, likely through high affinities for RNA and single-stranded DNA viral replication intermediates (Newman et al., 2005; Holmes et al., 2007; Shaban et al., 2018).

Follow-up studies revealed that Vif counteracts the mutagenic potential of the APOBEC3s by nucleating the formation of an E3-ubiquitin ligase complex that targets A3s for proteasomal degradation prior to virion encapsidation (Marin et al., 2003; Sheehy et al., 2003; Mehle et al., 2004). Proteomic and genetic studies determined that this E3-ubiquitin ligase complex is comprised of Cullin 5 (CUL5), Elongin B and C (ELOB/C), Ring-Box 2 (RBX2), and CBF-β (Yu et al., 2003; Jäger et al., 2011; Zhang et al., 2011; Figure 2C). Interestingly, CBF-β was not part of the original proteomics discovery, but instead, came several years later. Significant efforts were made by many labs to purify Vif from heterologous systems with little success, which led to the hypothesis that Vif may require a cellular co-factor to maintain stability. Quantitative proteomics experiments revealed that this co-factor was the transcription factor CBF-β, which co-precipitated with the E3-ubiquitin ligase complex only in the presence of Vif (Figure 2C, left). Further investigation established that CBF-β is indeed a *bona-fide* co-factor required for Vif expression and counteraction of APOBEC3 restriction factors *in vivo*, and that it is an integral part of the complex as evidenced by a high-resolution structure (Guo et al., 2014; Figure 2C).

## Dynamic Nature of Vif-Substrate Protein–Protein Interfaces

One of the major questions following the discovery of the Vif-APOBEC3 interaction was how can a relatively small viral protein (∼20 kDa) interact with four restrictive APOBEC3 enzymes (five in total), recruit CBF-β, nucleate the formation of an E3-ubiquitin ligase complex, and induce G2/M cell cycle arrest (discussed in further detail below)? Remarkably, it has become apparent that each Vif-substrate pairwise combination utilizes largely distinct, and genetically separable, interaction surfaces (Figure 2D). Importantly, these biochemical constraints suggest that a single Vif/E3-complex can only degrade one substrate at a time, making Vif’s ability to target so many distinct substrates even more impressive.

Extensive mutagenesis studies have identified several Vif amino acid substitutions that can disrupt individual pair-wise interactions and leave others intact. Distinct clusters of separation-of-function mutants have been identified that disrupt degradation of A3D/F, A3G, or A3H. The Vif surface utilized for A3G recognition is clustered near the N-terminus, encompassing three distinct sets of amino acid residues, with the ^40^YRHHY^44^ segment being unique to A3G recognition (Russell and Pathak, 2007; He et al., 2008; Yamashita et al., 2008; Chen et al., 2009; Dang et al., 2009; Pery et al., 2009; Letko et al., 2015; Figure 2D). While the A3D/F interface partially overlaps with A3G, it also extends to residues ^11^WQxDRMR^17^ and ^74^TGERxW^79^, which imparts specificity (Russell and Pathak, 2007; He et al., 2008; Zhang et al., 2008; Figure 2D). Interestingly, the surface residues used to recognize A3H are clustered at the top of the β-fold and are completely distinct from those used to recognize the other APOBEC3s (Zhen et al., 2012; Ooms et al., 2013, 2017; Nakashima et al., 2017; Wang et al., 2019). Likewise, the same is true for the Vif surfaces that maintain interactions with CBF-β (residues located on β-strand 1 and 6) (Zhou et al., 2012, 2014) and the E3-ubiquitin ligase complex (residues located on α-helix 3 and 4) (Kamura et al., 1998; Yu et al., 2004; Bergeron et al., 2010; Wolfe et al., 2010; Figure 2D).

Previous analyses of available Vif sequences have highlighted evolutionary conservation for some Vif-substrate surfaces but not others (Azimi and Lee, 2020). As one would predict, residues involved in binding CBF-β and the E3-ubiquitin ligase complex are highly conserved; however, the same is not true for residues involved in APOBEC3 recognition. The conservation of interface residues diminishes with A3F > A3G >> A3H, which is consistent with the current model that A3H may only be a threat to the virus in certain regions of the globe (Ebrahimi et al., 2018). This observation is also consistent with the “wobble model” which provides an evolutionary paradigm explaining the biochemical drift between Vif-APOBEC3 interactions (Richards et al., 2015; Harris and Anderson, 2016). This model is predicated on the concept that an ancestral lentivirus was established in a non-primate mammal with a relatively modest APOBEC3 gene-set prior to transmitting into a primate with an expanded APOBEC3 repertoire. In this hypothetical scenario, the strong interaction between Vif and the ancestral APOBEC3 is attenuated following zoonosis. The Vif-APOBEC3 interaction(s) are then restored following compensatory mutations encoding residues at, or near, the periphery of the ancestral Vif-APOBEC3 interaction surface. Importantly, these adaptations would have occurred independently for each novel APOBEC3 restriction factor, giving rise to the distinct interaction surfaces observed on present-day Vif (Figure 2D).

## Vif Induces G2/M Cell Cycle Arrest Through Degradation of PPP2R5s

Because counteraction of the APOBEC3s is critical for HIV-1 replication, this was thought to be Vif’s only function for many years. However, in the early 2000s it was discovered that Vif could also induce G2/M cell cycle arrest in a variety of cell lines (Sakai et al., 2006; Wang et al., 2007). Genetic studies established that Vif-induced G2/M arrest requires both CBF-β and the same E3-ubiquitin ligase complex utilized for APOBEC3 degradation (DeHart et al., 2008; Du et al., 2019). Additionally, fine-mapping of the Vif residues required for inducing G2/M arrest revealed that this interface is mostly distinct from those of the APOBEC3s (Izumi et al., 2010; Zhao et al., 2015; Salamango et al., 2019, 2020; Marelli et al., 2020; Nagata et al., 2020; Figure 2D). While these observations collectively pointed toward Vif degrading a cellular factor to induce arrest, the identity of this factor remained elusive for nearly 10 years following the discovery of this activity.

A major breakthrough regarding Vif-induced G2/M arrest came from recent quantitative proteomics studies that revealed Vif-dependent remodeling of the host phosphoproteome (Greenwood et al., 2016; Naamati et al., 2019). These studies discovered that Vif could efficiently deplete multiple members of the PPP2R5 family of protein phosphatase 2A (PP2A) regulators in several cell lines, including primary and immortalized CD4^+^ T cells (Greenwood et al., 2016; Naamati et al., 2019). PP2As account for a majority of the phosphatase activity in eukaryotic cells and function as heterotrimeric complexes comprised of a phosphatase enzyme (PP2Cα), a scaffolding protein (PPP2R1α or PPP2R1β), and a regulatory subunit (B55, PPP2R5/B56, or PR72/130) (Thompson and Williams, 2018; Nilsson, 2019). The regulatory subunit can be from one of three distinct protein families, which regulate subcellular localization and substrate recognition of PP2A holoenzyme complexes (McCright et al., 1996; Wang J. et al., 2016). Importantly, PP2A/PPP2R5 holoenzymes have been shown to regulate multiple aspects of the G2-to-M phase transition (Moura and Conde, 2019; Nilsson, 2019).

The connection between PP2A/PPP2R5 complexes and regulation of G2/M progression prompted our group, and others, to investigate if these were the factors antagonized by Vif to induce G2/M arrest. Comprehensive mutagenesis using both single amino acid substitutions and large-scale mutagenic libraries revealed that Vif residues required for degradation of PPP2R5 substrates overlap with those required for inducing G2/M arrest (Evans et al., 2018; Salamango et al., 2019, 2020; Marelli et al., 2020; Nagata et al., 2020). These studies also showed that siRNA-mediated knock-down of specific combinations of *PPP2R5* transcripts could induce a robust G2/M arrest phenotype in the absence of Vif; however, strong knock-down of single *PPP2R5* family members had no impact on cell cycle progression (Salamango et al., 2019; Marelli et al., 2020). While subtle differences were observed for which combinations of *PPP2R5* knock-downs were most impactful, the overall conclusions were complementary in that at least two family members need to be simultaneously depleted to induce arrest in the absence of Vif (Salamango et al., 2019; Marelli et al., 2020). Additionally, chemical inhibition of PP2A activity using okadaic acid induced a robust G2/M arrest phenotype, further supporting a direct relationship between Vif-induced PPP2R5 degradation and subsequent G2/M arrest (Salamango et al., 2019; Marelli et al., 2020).

## Vif Binds the Same Surface as Physiologic PPP2R5 Substrates

**FIGURE 3:**
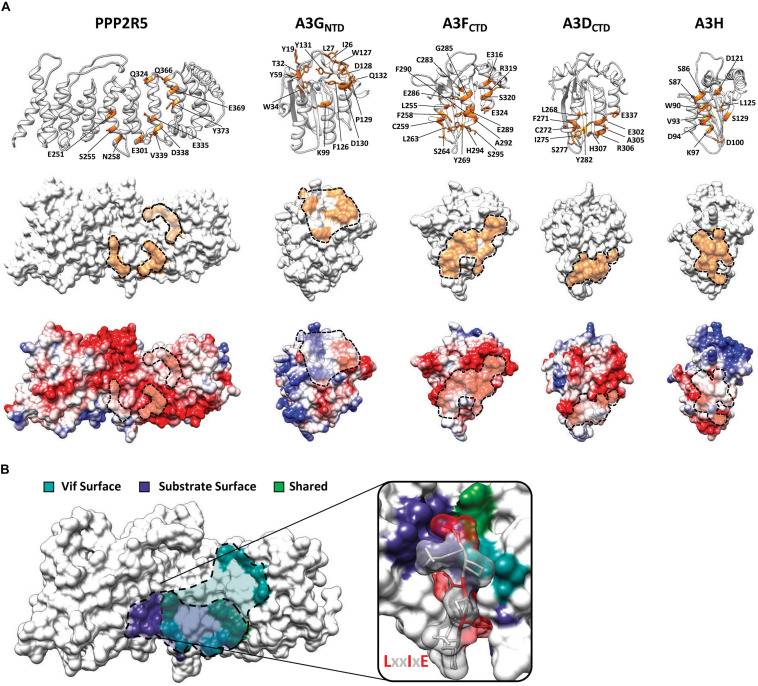
Substrate surfaces recognized by Vif. **(A)** Amino acid residues on PPP2R5 and APOBEC3 that have been shown to be required for Vif-mediated degradation (top) with substrate surfaces (middle) and electrostatic potential maps (bottom). **(B)** Depiction of amino acid residues on the surface of PPP2R5 that are recognized by Vif or cellular substrates. The LxxIxE substrate peptide is depicted bound to the PPP2R5 surface to highlight that Vif and cellular substrates compete for an overlapping surface.

Characterization of Vif separation-of-function mutants suggested that PPP2R5 recognition occurs through electrostatic interactions, as a majority of residues required for degradation are positively charged (8 out of 12 residues) (Salamango et al., 2019, 2020; Marelli et al., 2020; Figure 2D). Using high-resolution crystal structures and homology modeling, it is evident that all Vif-substrate interaction surfaces are largely electrostatic in nature (Kitamura et al., 2012; Li et al., 2012; Bohn et al., 2013; Byeon et al., 2013; Siu et al., 2013; Wang J. et al., 2016; Kouno et al., 2017; Shi et al., 2017; Ito et al., 2018; Shaban et al., 2018; Figure 3A). The PPP2R5 surface recognized by Vif is extremely electronegative and supports a model in which Vif maintains this interaction through a favorable network of electrostatics. Importantly, all of the residues required for Vif recognition are conserved among all five family members, which clarifies how Vif can recognize and degrade five new cellular substrates and explains genetic evidence that loss of at least two family members is required for inducing G2/M arrest.

Interestingly, the PPP2R5 surface recognized by Vif overlaps with the substrate binding pocket used by PPP2R5s to bind cellular substrates (Hertz et al., 2016; Wang J. et al., 2016; Wang X. et al., 2016; Figure 3B). Crystallographic and proteomics studies have combined to identify the substrate binding motif recognized by PPP2R5 proteins and the mode in which binding occurs. PPP2R5s recognize substrates through a conserved LxxIxE motif that directs the PP2A holoenzyme complex to cellular targets for dephosphorylation (Hertz et al., 2016; Wang J. et al., 2016; Wang X. et al., 2016). The LxxIxE motif is stabilized by two distinct substrate binding pockets on the surface of PPP2R5 proteins. The “Leu pocket,” comprised of residues K208, T184, H187, R188, E251, and I227, and the “Ile pocket,” which contains residues H187, Y215, I227, and I256. In addition, a recent study demonstrated that PPP2R5 substrate recognition is enhanced by electrostatic interactions that occur adjacent to the peptide binding cleft, encompassing residues E335 and D338 (Wang X. et al., 2020). These residues are of interest because E251, E335, and D338 are required for Vif-induced degradation of PPP2R5s (Salamango et al., 2019; Figure 3B). Furthermore, residues S255 and N258, which are located within the “Ile pocket,” are also required for Vif-induced degradation (Figure 3B).

The close proximity and partial overlap between these interfaces raised the question of whether Vif and cellular substrates compete for binding. To test this model, we utilized a well-characterized peptide inhibitor that contains the LxxIxE motif recognized by PPP2R5s to determine if Vif-induced degradation could be blocked. Co-transfection of a plasmid expressing four tandem copies of this LxxIxE peptide caused a dose-dependent inhibition of Vif-mediated degradation of PPP2R5A (Salamango et al., 2020). However, a control plasmid containing an AxxAxA motif had no effect on Vif-induced degradation of either PPP2R5A or APOBEC3G (Salamango et al., 2020). Taken together, these observations support a competitive binding model in which Vif directly interacts with the surface of PPP2R5 proteins and occludes the binding of cellular substrates.

## Mechanistic Model for Vif-Induced G2/M Arrest

The discovery of PPP2R5 substrates was a major step forward in understanding Vif-induced cell cycle arrest. However, the regulatory checkpoints altered downstream of PPP2R5 degradation remain unknown. PP2A/PPP2R5 complexes have been shown to regulate entrance and exit of the G2-to-M phase transition at multiple different checkpoints. Below, we discuss these checkpoints in the context of previous observations regarding Vif-induced G2/M arrest and postulate a model in which antagonism of discrete PP2A/PPP2R5 complexes would lead to simultaneous inhibition of multiple checkpoints.

**FIGURE 4:**
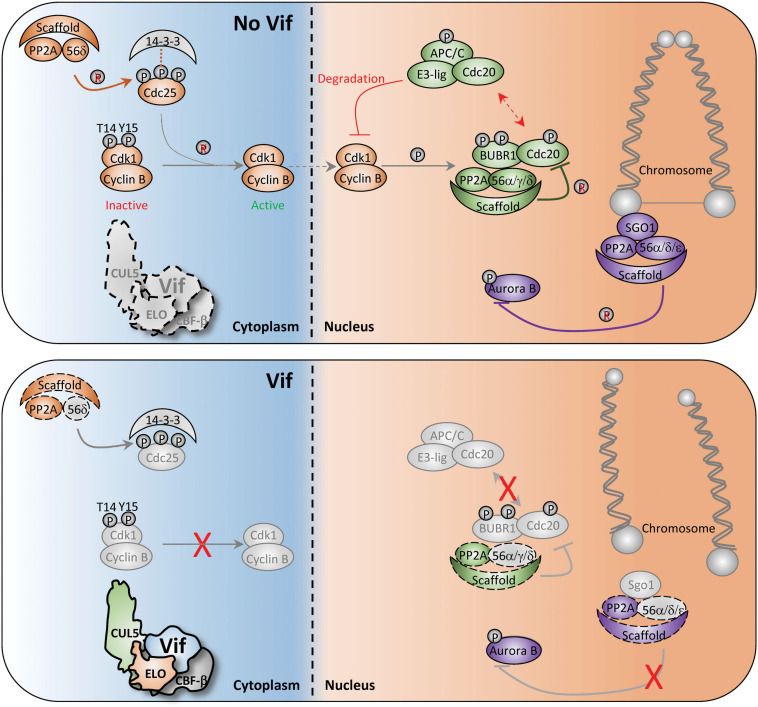
Diagram of potential Vif-induced G2/M arrest mechanisms. Depiction of normal and aberrant regulation of the G2-to-M phase transition in the absence (top) and presence (bottom) of Vif. Key cell cycle checkpoints regulated by PP2A/PPP2R5 complexes are color-coded in the absence or presence of Vif. See text for additional details.

First, arguably the most critical event for mitotic entry is CDC25-mediated activation of CDK1, which allows for nuclear translocation of the CDK1-CyclinB complex (Lee and Kirschner, 1996; Timofeev et al., 2010; Figure 4, top). Activation of CDC25 requires PP2A-mediated dephosphorylation of specific residues to release inhibitory 14-3-3 proteins (Margolis et al., 2006; Figure 4, top). Therefore, Vif-induced antagonism of PP2A/PPP2R5 complexes would render CDC25 inactive and inhibit nuclear translocation of CDK1-CyclinB1 (Figure 4, bottom). In support of this mechanism, a previous study reported that infection of Jurkat T cells with arrest-proficient HIV-1 resulted in constitutive phosphorylation of Tyr15 on CDK1, and subsequent inhibition of CDK1-CyclinB nuclear translocation (Sakai et al., 2011). Additionally, quantitative proteomics and immunoblot analyses revealed elevated levels of CyclinB in CEM T4 cells infected with HIV-1 expressing arrest-proficient Vif (Marelli et al., 2020).

Second, PP2A/PPP2R5 complexes also control G2-to-M progression through spatiotemporal regulation of the Aurora kinases. Aurora kinases are involved in numerous G2-to-M checkpoint controls including spindle assembly, microtubule-kinetochore attachment, and cytokinesis (Fu et al., 2007; Goldenson and Crispino, 2015). To exit mitosis, Aurora kinases are degraded through the APC-ubiquitin-proteasome pathway, which is regulated by PP2A/PPP2R5A, 5C, and/or 5D complexes (Horn et al., 2007; Jeong and Yang, 2013; Lindon et al., 2015; Figure 4, top). Recent studies have demonstrated striking activation of Aurora kinase A and B following infection of CEM T4 cells with HIV-1 expressing arrest-proficient Vif, supporting a model in which Vif simultaneously antagonizes PP2A/PPP2R5 complexes to inactivate the APC system and stall mitotic exit (Figure 4, bottom).

Lastly, PP2A/PPP2R5 holoenzymes have been shown to serve as a scaffold for multiple distinct complexes to initiate exit from mitosis. For example, Shugoshin (SGO1) directly binds to a PP2A/PPP2R5 holoenzyme to form a complex that regulates accurate chromosome segregation during mitosis (Tang et al., 2006; Xu et al., 2009; Figure 4, top). Without the formation of this complex, centromeric cohesion is prematurely cleaved which results in untimely centromere disassociation and chromosome mis-segregation (Tang et al., 2006; Xu et al., 2009; Figure 4, bottom). Additionally, PP2A/PPP2R5 holoenzymes directly bind to BUBR1 and regulate the spindle assembly checkpoint and mitotic progression by counteracting Aurora kinase activity at improperly attached kinetochores (Cheeseman et al., 2006; DeLuca et al., 2006; Kruse et al., 2013). Therefore, disruption of BUBR1 regulatory activity and hyper-activation of Aurora kinases through Vif-mediated antagonism of PP2A/PPP2R5 complexes would result in kinetochore dysfunction and a failure to exit mitosis (Figure 4, bottom).

In addition to manipulation of the PP2A/PPP2R5 axis, it has been suggested that Vif can induce G2/M arrest by blocking MDM2-mediated ubiquitination and nuclear export of TP53 (Izumi et al., 2010). This study observed that Vif binding to TP53 could block MDM2 recognition and subsequent turnover of TP53, which is required for proper cell cycle progression. Although not addressed directly, it is likely that the Vif/TP53 interaction occurs in the nuclear compartment, thus directly shielding TP53 from MDM2 recognition and subsequent nuclear export. If this is indeed the case, it is plausible that there are two pools of Vif that act in concert to stall cell cycle progression. Nuclear localized Vif protects TP53 from MDM2 and antagonizes nuclear PPP2R5C/D substrates, whereas cytoplasmic Vif antagonizes the APOBEC3s and cytoplasmic PPP2R5A/B/E. Additional work will be required to determine if these mechanisms are separate or connected through shared components.

## Concluding Remarks

Now, Vif clearly has two distinct sets of cellular substrates, APOBEC3 enzymes and PPP2R5 phospho-regulators (10 proteins total). While APOBEC3 counteraction has been shown to be essential for viral infectivity and pathogenesis *in vivo*, the importance of PPP2R5 degradation remains to be established. However, at least three key observations have been made that imply that alteration of the cell cycle may be beneficial for HIV-1 pathogenesis.

First, HIV-1 Vpr also potently induces G2/M arrest through a mechanism that is clearly distinct from that of Vif. Vpr activates the ATR DNA damage response pathway by inducing proteasomal degradation of diverse cellular substrates using a Cullin 4 (CUL4), DNA damage-binding protein 1 (DDB1), and DDB1-CUL4-associated factor (DCAF1) E3-ubiquitin ligase complex (Hrecka et al., 2007; Andersen et al., 2008; Greenwood et al., 2019). While Vif degrades a smaller set of cellular proteins, Vpr has been shown to be much more promiscuous. A recent study demonstrated that Vpr induces the degradation of roughly 40 cellular proteins causing a systems-level remodeling of the cellular proteome, which may cumulatively lead to G2/M arrest (Greenwood et al., 2019). Collectively, over half a dozen cellular proteins have been directly implicated as being essential for Vpr-induced arrest [e.g., MCM10, SMN1, CDCA2, ZNF267, MUS81, CCDC137, etc. (Laguette et al., 2014; Romani et al., 2015; Greenwood et al., 2019; Zhang and Bieniasz, 2020)].

At least two phenotypes have been associated with Vpr-induced arrest that may enhance HIV-1 pathogenicity. First, degradation of MUS81 and EME1 by Vpr leads to G2/M arrest and prematurely activates the SLX4 complex, which suppresses spontaneous and HIV-1-mediated induction of type I interferon responses and may contribute to immune evasion (Laguette et al., 2014). Second, depletion of CCDC137 by Vpr has been shown to induce G2/M arrest and enhance HIV-1 gene expression (Zhang and Bieniasz, 2020). It is worth emphasizing that during the G2/M phase of the cell cycle, cellular translation and transcription are significantly diminished (Parsons and Spencer, 1997; Kronja and Orr-Weaver, 2011; Tanenbaum et al., 2015; Strzyz, 2017). Therefore, Vpr’s ability to simultaneously induce arrest and enhance HIV-1 gene expression would lead to an enrichment of viral transcripts during G2/M. Interestingly, PP2A/PPP2R5 complexes have been shown to regulate protein translation kinetics through mTOR, S6 kinase, and 4E-BP1 (Hahn et al., 2010; Gardner et al., 2015). Therefore, it is possible that Vif antagonism of PP2A holoenzymes leads to increased protein translation during G2/M when host cell translation is normally stalled. Taken together, it is possible that Vif and Vpr act in concert to induce G2/M arrest and boost transcription and translation of HIV-1 genes.

Second, arrest-proficient Vif variants are prominent in global populations and in patient derived Vif isolates. Bioinformatic analyses of sequences obtained from the Los Alamos database have drawn correlations between predicted arrest-proficient Vif sequences and the most abundant subtypes in global circulation (Salamango et al., 2019; Marelli et al., 2020). Additionally, a previous study suggested that Vif-induced arrest leads to increased production of HIV-1 particles from both primary and immortalized CD4^+^ T cells (Izumi et al., 2010). Although this is the only study to-date that demonstrates this phenotype, these findings could explain why arrest-associated Vif variants are prevalent in circulating populations. Furthermore, live cell imaging studies assessing degradation kinetics of APOBEC3G and PPP2R5A in the same cell established that the half-life of PPP2R5A is only ∼2 h slower than that of APOBEC3G following infection, which is striking given that APOBEC3G degradation is essential for HIV-1 infectivity and pathogenesis (Salamango et al., 2020).

Third, subversion of the cell cycle is a mechanism used by diverse viral pathogens to create a favorable environment for replication. Oncogenic viruses, RNA viruses, and DNA viruses have all been shown to hijack the host cell cycle during the course of an infection, many of which arrest cells in the G2/M phase [e.g., human T-lymphotropic virus (HTLV), human polyoma virus, infectious bronchitis virus, simian virus 40, and adenovirus (Zhao and Elder, 2005; Dove et al., 2006; Fan et al., 2018)]. Interestingly, PP2A appears to be a conserved target as well, given that simian virus 40, polyoma virus, HTLV, adenovirus, and HIV-1 affect the enzymatic activity of at least a subset of PP2A complexes (Roopchand et al., 2001; Haoudi et al., 2003; Zhao and Elder, 2005). Importantly, PPP2R5 proteins have also been shown to be hijacked by other viral pathogens. Ebola virus nucleoprotein recruits PPP2R5 proteins through a highly conserved LxxIxE motif to stimulate dephosphorylation of VP30, which initiates transcription of the viral genome and subsequent infection (Kruse et al., 2018). Additionally, HTLV requires PPP2R5C for efficient strand-transfer activity and target integration (Maertens, 2016). HTLV integrase uses a LxxIxE motif to recruit PPP2R5C to the viral integration complex (Bhatt et al., 2020). Taken together, these observations support a model in which PPP2R5 antagonism, and global changes in the cellular phospho-proteome, are likely to be advantageous for the pathogenesis of HIV-1 as well as other prominent viruses. Furthermore, continuing to unravel the complex molecular mechanisms HIV-1 has evolved to subvert cellular processes and enhance pathogenicity may provide major clues for the development of innovative therapeutics that lead to virus eradication.

## Author Contributions

DS drafted the manuscript. DS and RH edited the manuscript. Both authors contributed to the article and approved the submitted version.

## Conflict of Interest

RH is a co-founder, shareholder, and consultant of ApoGen Biotechnologies Inc. The remaining author declares that the research was conducted in the absence of any commercial or financial relationships that could be construed as a potential conflict of interest.
